# Investigating the effects of differently produced synthetic amorphous silica (E 551) on the integrity and functionality of the human intestinal barrier using an advanced in vitro co-culture model

**DOI:** 10.1007/s00204-020-02957-2

**Published:** 2020-12-15

**Authors:** Claudia Hempt, Cordula Hirsch, Yvette Hannig, Alexandra Rippl, Peter Wick, Tina Buerki-Thurnherr

**Affiliations:** 1 Laboratory for Particles-Biology Interactions, Empa, Swiss Federal Laboratories for Materials Science and Technology, Lerchenfeldstrasse 5, 9014 St. Gallen, Switzerland; 2 Department of Health Sciences and Technology, ETH Zürich, Zürich, Switzerland

**Keywords:** Synthetic amorphous silica (E551), Intestinal co-cultures, In vitro toxicology, Structure–activity relationships, Intestine-specific functional endpoints

## Abstract

E 551, also known as synthetic amorphous silica (SAS), is the second most produced food additive. However, according to the re-evaluation of E 551 by the European Food Safety Authority (EFSA) in 2018, the amount of available data on the oral toxicity of food grade E 551 is still insufficient for reliable risk assessment. To close this gap, this study aimed to investigate six food-grade SAS with distinct physicochemical properties on their interaction with the intestinal barrier using advanced in vitro intestinal co-cultures and to identify potential structure–activity relationships. A mucus-secreting Caco-2/HT-29/Raji co-culture model was treated with up to 50 µg/ml SAS for 48 h, which represents a dose range relevant to dietary exposure. No effects on cell viability, barrier integrity, microvilli function or the release of inflammatory cytokine were detected after acute exposure. Slight biological responses were observed for few SAS materials on iron uptake and gene expression levels of mucin 1 and G-protein coupled receptor 120 (GPR120). There was no clear correlation between SAS properties (single or combined) and the observed biological responses. Overall, this study provides novel insights into the short-term impact of food-relevant SAS with distinct characteristics on the intestinal epithelium including a range of intestine-specific functional endpoints. In addition, it highlights the importance of using advanced intestinal co-cultures embracing relevant cell types as well as a protective mucus barrier to achieve a comprehensive understanding of the biological response of food additives at the intestinal barrier in vitro.

**Electronic supplementary material:**

The online version of this article (10.1007/s00204-020-02957-2) contains supplementary material, which is available to authorized users.

## Introduction

Over the last years, the European Food Safety Authority (EFSA) has re-evaluated many food additives (EFSA, 2015, 2016a, b, c, d, 2017a, b, 2018a, b). This included synthetic amorphous silica (SAS), which are defined as nanostructured materials (Bosch et al. 2012). They are registered in the European Union under E 551 (European Commission 2008). SAS are processing agents, which can be added to food and non-food products as an anti-caking agent, flow aid, clearing agent or stabiliser (EU 2008; OECD 2004; Van Kesteren et al. 2015).

A considerable amount of toxicity assessments of silica materials has already been performed in in vitro as well as in animal models with slightly varying outcomes (Fruijtier-Pölloth 2016). An *in vivo* study in rodents indicated that oral exposure to food-grade SAS (E 551) did not induce systemic toxicity or immunotoxicity (Van der Zande et al. 2014). In contrast, fumed silica induced cytotoxic responses in bronchial epithelial cells and macrophages in vitro (Zhang et al. 2012). It is often difficult to compare different studies and to draw firm conclusions on the safety of food grade SAS as many of these toxicity studies do not specify what type of silica has been used, and even silica materials that are not authorized as E 551 were included in the evaluation (Fruijtier-Pölloth, 2016; Sohal et al. 2018). It has been pointed out by the European Food Safety Authority (EFSA) as well as Maynard (2014) that more data are needed to fully evaluate SAS (EFSA, 2018a). The EFSA hinted that the unique characteristics of the different SAS forms (precipitated, gel, pyrogenic and colloidal, while the colloidal is not authorized as E 551) may alter their behaviour (EFSA, 2018a). For instance, Zhang et al. (2012) found a higher toxicity for fumed compared to colloidal silica and postulated that this was due to the presence of siloxane rings in fumed silica. Therefore, the use of food-grade SAS and a comprehensive characterisation of the used materials is pivotal to identify structure–activity relationships. Moreover, Sohal et al. (2020) suggested to perform a full toxicity evaluation of SAS in vitro in advanced intestinal co-culture models and to cover not only conventional toxicity endpoints (e.g., cell viability, oxidative stress and pro-inflammatory responses) but also address potential functional changes of the intestinal barrier. Endpoints to assess physiological functions of the intestine in vitro have only recently started to be investigated for TiO_2_ and SiO_2_ nanomaterials such as the uptake of iron, glucose or lipids (Guo et al. 2018, 2017). Moreover, a recent paper on the translocation of E 551 in an in vitro intestinal model suggested different intestinal transport mechanism for E 551 and nano-sized SiO_2_ resulting in an overall transport of 1.4–1.6% for E 551 and 3.6% for nano-sized SiO_2,_ respectively (Yu et al. 2020).

A prevalent 2D model to study toxicity at the intestinal barrier is the human colorectal adenocarcinoma Caco-2 cells, which form a polarised epithelial monolayer with a microvilli brush border on a semipermeable insert (Jumarie and Malo 1991). However, differentiated Caco-2 monolayers poorly represent the complex anatomical and physiological situation of the intestine and lack the mucus layer, which constitutes an important mechanical and chemical barrier (Chopra et al. 2010).

The improvement of intestinal cultures by including multiple relevant human intestinal cell types as well as a confluent mucus layer presents a promising approach towards a more predictive in vitro toxicity assessment of food relevant materials. In the recent years, intestinal organoid culture (Brandenberg et al. 2020; Sato et al. 2009) and highly advanced co-cultures (Antunes et al. 2013; García-Rodríguez et al. 2018a; Schimpel et al. 2014) have been established. The advanced co-cultures have been successfully developed to express a mucus barrier and/or contain the most common cell types of the intestine (e.g., enterocytes, goblet cells, M-cells) (Antunes et al. 2013; García-Rodríguez et al. 2018a; Schimpel et al. 2014). Indeed, incorporation of M-cells has been proven to be critical to achieve predictive results for nanomaterial uptake since the fate of nanomaterials in the gastro intestinal tract (GIT) is very much driven by these cells (Powell et al. 2010). These advanced intestinal co-cultures could also be very useful to assess the toxicity of food compounds and additives on viability, integrity and physiological function of the intestinal barrier. However, suitable assays need to be identified that are compatible with these advanced co-cultures, in particular since the presence of a dense mucus layer could interfere with conventional toxicity assays.

In a previous study, we have investigated the deposited dose and acute toxicity of 10 food-grade SAS materials of commercial relevance in differentiated Caco-2 monolayers (Hempt et al. 2020). We did not observe any considerable acute toxicity of the different SAS materials independent of the production process and the material properties. This type of studies, including relatively simple monoculture models and few acute toxicity endpoints, are interesting for a first screening of a large variety of (nano)materials. However, the use of more advanced intestinal co-culture models and inclusion of sensitive functional cellular assays is pivotal to achieve a more comprehensive understanding of (nano)material interactions at the intestinal barrier. Here, we applied an advanced in vitro intestinal model (Caco-2/HT-29-MTX-E12/Raji B) with a confluent mucus layer and M-cells to investigate the impact of six food-grade SAS materials to identify potential structure–activity relationships. These six nanostructured materials differ in aggregate size, surface area, silanol content and production route. This study especially focused on the evaluation of intestine-specific functional effects like mucus coverage, microvilli function, lipid uptake, iron uptake and barrier integrity besides more conventional viability and inflammatory responses.

## Material and methods

### Particle synthesis, dispersion and characterisation

Six food grade SAS materials were kindly provided by Evonik Operations GmbH (Hanau, Germany). These included precipitated silica produced by wet process (SIPERNAT® materials) as well as fumed silica produced by flame hydrolysis (AEROSIL® materials) (EFSA 2018a; IPTS/EC 2007). An extensive characterisation of these SAS has been performed in our previous publication (Hempt et al. 2020) and a summary of the particle characteristics is provided in Table 1.

**Table 1:** SAS characterisation

Material	Production process	Primary structure size/nm^I^	Aggregate size (ECD)/nm^II^	Specific surface area/ (m^2^/g)^III^	Material density/ (g/cm^3^)^IV^	Agglomerate effective density/ (g/cm^3^)^V^	Agglomerate size/ nm^VI^	Point of zero charge/ pH^VII^	Zeta potential/ mV^VIII^	Silanol content/ (Si–OH/nm^2^) ^IX^
SIPERNAT^®^ 350	Precipitated	30.3 ± 6.8	276.3	55	2.15	1.33	623±15	1.7	n.a	3.0–3.2
SIPERNAT^®^ 22 S	Precipitated	10 ± 2.6	82.2	185	2.14	1.20	743±38	2.0	− 37.1	3.4–3.6
SIPERNAT^®^ 160	Precipitated	12.2± 2.7	58.3	180	2.16	1.18	555±32	1.7	− 41.2	3.4–3.6
SIPERNAT^®^ 50 S	Precipitated	3.1± 0.7	59.8	460	2.12	1.16	747±86	1.7	− 13.7	3.4–3.6
AEROSIL^®^ OX50	Fumed	41.4± 18.3	233.7	45	2.32	1.25	299±6	2.3	− 34.0	1.4–1.5
AEROSIL^®^ 380 F	Fumed	8 ± 2.7	101.9	390	2.29	1.09	318±15	2.4	− 10.6	1.7–1.8

The materials were characterised and used in a previous study (Hempt et al. 2020) and a summary is provided here. (I/II) Primary structure size and aggregate size as assessed by TEM, arithmetic average ± SD; *ECD* equivalent circular diameter. (III) Specific surface area assessed by N_2_-BET. (IV) Material density assessed by gas displacement pycnometry. (V) Agglomerate effective density assessed by volumetric centrifugation method from 0.1 mg/ml dispersion in ddH_2_O, (VI) Agglomerate size assessed by dynamic light scattering after Ultra Turrax T25 dispersion in ddH_2_O. (VII/VIII) point of zero charge and zeta potential measured with an electroacoustic sensor at a solid density of 2.1 g/ml. (IX) silanol content determined by lithium alanate titration

Surface hydroxyl concentrations of SAS were provided by Evonik Operations GmbH (Hanau, Germany) and were estimated by reaction with lithium aluminium hydride (lithiumalanate, LiAlH_4_). Briefly, hydrogen evolving by reaction of LiAlH_4_ with the surface hydroxyl groups (see Eq. 1) was measured and the silanol density was calculated according to Eq. 2:

1 $$ 4{\text{~}} \equiv {\text{~Si~}} - {\text{~OH~}} + {\text{~LiAlH}}_{4}  \to {\text{~}} \equiv {\text{~Si~}}{-}{\text{~O~}} - {\text{~Li~}} + {\text{~}}\left( { \equiv {\text{~Si~}} - {\text{~O}}} \right)_{3} {\text{Al~}} + {\text{~}}4{\text{~H}}_{2}  $$

2 $$ {{{\text{OH}}} \mathord{\left/ {\vphantom {{{\text{OH}}} {{\text{nm}}^{2} }}} \right. \kern-\nulldelimiterspace} {{\text{nm}}^{2} }} = ~\frac{{\frac{{{\text{V}}_{{{\text{H2}}}} {\text{~}} \times {\text{p}}}}{{{\text{R~}} \times {\text{T}}}}{\text{N}}_{{\text{A}}} }}{{{\text{10}}^{{{\text{24}}}} {\text{~}} \times {\text{~SSA~}} \times {\text{~m~}}}}, $$

where *V*_*H2*_ is the hydrogen volume (ml), *p* the pressure (Pa), T the temperature (K), SSA the specific surface area (m^2^/g) and m the sample mass (g). N_A_ and R are the Avogadro and the ideal gas constant, respectively.

No acid pre-treatment was performed since it has been shown that it did not alter the characteristics of the SAS materials (Maier et al. 2013).

Stock dispersions of SAS (10 mg/ml) were prepared as previously described (Hempt et al. 2020) in double distilled water (ddH_2_O) using an ULTRA-TURRAX^®^ T25 (IKA, Staufen, Germany) at 14,600 rpm for 1 min, which results in a particle size distribution found in food matrix (Contado et al. 2013; Maier et al. 2015 as cited in EFSA et al. 2018). Stock dispersions were stored at room temperature (RT) for up to 12 weeks and treated again with ULTRA-TURRAX^®^ prior to any SAS treatment.

### Cell culture

The human colorectal adenocarcinoma cell line Caco-2 was obtained from the German collection of microorganisms and cell cultures (DSMZ, Braunschweig, Germany). Cells were maintained in Minimum Essential Medium (MEM) (Sigma) supplemented with 10% fetal calf serum (FCS), 2 mM L-glutamine, 1% (v/v) penicillin, streptomycin, neomycin (PSN) and 1% (v/v) non-essential amino acids (all from Sigma) (hereafter called “complete MEM") at 37 °C, 5% CO_2_ and 95% humidity, hereafter called “standard growth conditions”. Caco-2 cells were grown in 75 cm^2^ cell culture flasks (TPP) until reaching 80% confluence and subcultured using trypsin–EDTA (Sigma). The human colon cell line HT-29-MTX-E12 (hereafter called “HT-29”) was obtained from the European Collection of Authenticated Cell Cultures (ECACC, England). HT-29 were maintained in Dulbecco’s Modified Eagle Medium (DMEM) (Sigma), supplemented with 10% FCS, 2 mM l-glutamine, 1% (v/v) PSN and 1% (v/v) non-essential amino acids (all from Sigma) (hereafter called “complete DMEM”) at standard growth conditions. HT-29 cells were grown in 75 cm^2^ cell culture flasks (TPP) until reaching 80% confluence and subcultured using trypsin–EDTA (Sigma). The human B lymphocyte cell line Raji (ATCC^®^ CCL-86™) was obtained from ATCC (LGC Standards GmbH, Wesel, Germany). Raji cells were maintained in RPMI-1640 (Seroglob), supplemented with 10% FCS and 1% (v/v) PSN (all from Sigma) (hereafter called “complete RPMI”) at standard growth conditions. Raji cells were grown in 75 cm^2^ cell culture flasks (TPP) for 4 days and subcultured according to the cell bank protocol.

### Establishment of advanced intestinal co-cultures

**Fig. 1:**
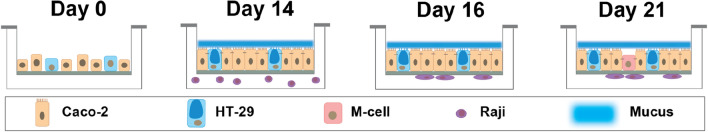
Schematic of the cultivation of the advanced intestinal co-culture model

The advanced intestinal co-culture model is based on co-culturing Caco-2 and HT-29 cells with a predefined seeding ratio of 75:25 to achieve a confluent mucus layer. An overall cell seeding concentration of 2 × 10^4^ cells/mm^2^ for the apical compartment of different microporous membrane inserts (Corning^®^ HTS Transwell^®^*-*96 Tissue Culture Systems (pore size: 3 µm), ThinCert™ Tissue Culture Inserts 12-Well Greiner Bio-One (pore size: 3 µm) or ThinCert™ Tissue Culture Inserts 6-Well Greiner Bio-One (pore size: 3 µm)) was maintained. The apical and the basolateral compartment were filled with complete DMEM and cells were allowed to differentiate under standard growth conditions for 14 days (Fig. 1). Medium was changed every other day. The addition of Raji B lymphocytes to Caco-2 cells allows differentiation of Caco-2 cells to M-cells (des Rieux et al. 2007). At day 14 5 × 10^5^ Raji cells per Transwell^®^ area of 113.1 mm^2^ were seeded in complete RPMI in the basolateral compartment. Similar to previous protocols Raji B lymphocytes were added to the basolateral compartment (Antunes et al. 2013; Brun et al. 2014; Lee et al. 2017; Mahler et al. 2012; Yu et al. 2020) and co-cultured for 48 h. At day 16 the Raji B lymphocytes were taken out of the basolateral compartment and advanced co-cultures were cultured for additional 5 days (total differentiation period: 21 days). Medium (apical: complete DMEM, basolateral: complete RPMI) was changed every other day.

### Treatment of advanced intestinal co-cultures with SAS

Treatment volumes in the apical compartments of 6-, 12- and 96-well inserts were calculated to match medium heights of 5.2 mm in all well sizes in order to achieve identical dosing conditions. Therefore, the volume of SAS dispersions added to the apical side was 2.38 ml for 6-well inserts, 590 µl for 12-well inserts and 75 µl for HTS 96-well inserts. Basolateral volumes were 2 ml for 6-well inserts, 1.5 ml for 12-well inserts and 235 µl for HTS 96-well inserts. Cells were treated in complete cell culture medium made from phenol red free DMEM (Gibco).

### Cell viability (MTT assay)

Advanced intestinal co-cultures in HTS 96-well inserts were treated in the apical compartment with 0, 3.125, 6.25,12.5, 25 or 50 µg/ml of SAS (for SIPERNAT^®^ 22 S a higher concentration of 200 µg/ml was included) for 24 h and 48 h. Amine modified polystyrene nanoparticles (PS-amine) (Bangs Laboratories, PA02N, Indiana, USA) were used as a particle positive control (Xia et al. 2006, 2008) at concentrations of 0,12.5, 25, 50, 100 and 200 µg/ml and added apically for 24 h and 48 h. The chemical positive control cadmium sulphate (CdSO_4_, Sigma) was added to the cells from the basolateral side in a concentration range from 0, 1, 10, 100, 1000 and 10,000 µM. The basolateral compartment for all other treatments was filled with complete DMEM medium made from phenol red free medium (Gibco). All 0 µg/ml samples received an equivalent volume of water and served as solvent control. The MTT stock solution (5 mg/ml in PBS; Sigma, M5655) was diluted in complete DMEM made from phenol red free medium. 24 h and 48 h post-exposure the wells were washed twice with warm PBS before adding 0.5 mg/ml MTT. In the apical compartment a volume of 75 µl/well and in the basal compartment of 235 µl/well was added. The cells were incubated for 1.5 h at standard growth conditions. Afterwards cells in the apical compartment were lysed through the addition of 37.5 µl 10% SDS in 0.01 M HCl, the basal compartment was exchanged to an empty one. After overnight efficient lysis of the cell layer 60 µl of the lysate was transferred to a standard 96-well plate and the absorbance was measured with a multi-well plate reader (Mithras^2^ LB943, Berthold Technologies) at 590 nm. Interference analysis was performed at three different concentrations of SAS (0.05, 3.13 and 50 µg/ml) in one independent experiment with 2 technical replicates. None of the SAS quenched the formazan signal, reduced MTT to formazan or exhibited auto-absorption up to a concentration of 50 µg/ml (data not shown).

### Measurement of transepithelial electrical resistance (TEER)

The differentiation process of the advanced intestinal co-cultures grown in 12- and 6-well inserts was evaluated by TEER measurements. The differentiation process was traced for one set of samples after every medium change and for all other cultures after 7, 14, 16 and 21 days using an Epithelia Voltohmmeter (EVOM) with sterilised STX2 electrodes (World precision, Instruments, Sarasota Florida, US). Only co-cultures with TEER values higher than 350 Ωcm^2^ were used for further experiments. The influence of SAS and PS-amine treatment on TEER values was evaluated after 48 h right before the 12-well inserts were processed for subsequent analysis.

### Stainings

#### Alcian blue staining

For the investigation of the mucus layer, an alcian blue staining was conducted. The 12-well inserts were fixed for 2 h with modified Carnoy solution (60% EtOH, 30% chloroform; 10% acetic acid) to preserve the mucin layer after 48 h of treatment of the advanced co-cultures with 50 µg/ml SAS. For long-term storage the inserts were immersed in 50% EtOH and kept at 4 °C. The inserts were stained with Alcian Blue 8GX (Sigma, 05,500) for 30 min before they were dehydrated and dried. For measurement in the plate reader the inserts were cut out of the insert holder and placed into a 24-well plate. Finally, absorbance was measured for 25 areas (5 × 5 µm each) per insert at 595 nm wavelength in a multi well plate reader (ELx800, BioTEX).

#### Immunocytochemistry

Advanced intestinal co-cultures in 12-well inserts after 21 days of culture were washed twice with PBS and fixed with 4% paraformaldehyde for 1 h at RT. The cells were washed with PBS and incubated with 30% sucrose at 4 °C overnight. Thereafter, cells were permeabilised with 0.1% Triton X-100 for 15 min, washed three times with PBS and non-specific binding sites were blocked for 30 min with 5% bovine serum albumin (BSA) in PBS. Cells were incubated with Alexa Fluor^®^ 488 conjugated mouse anti-ZO-1 antibody (Invitrogen 339,188; 1:50), mouse anti-MUC5AC antibody (Sigma; M5293; 1:100) or rat anti-NKM 16–2-4 antibody (Miltenyi Biotec; 130–096-148; 1:100) in 1% BSA in PBS at 4 °C overnight. After three washing steps in PBS, cells were incubated accordingly for 1 h with goat anti-rabbit Alexa Fluor® 555 (Invitrogen; A21428; 1:400), goat anti-mouse Alexa Fluor^®^ 488 (Invitrogen; A11029; 1:400) or goat anti-rat Alexa Fluor® 555 (Invitrogen; A21434; 1:400) and phalloidin Alexa Fluor 633 (Invitrogen A22284; 1:50) in 1% BSA in PBS. After additional three washing steps in PBS, nuclei were counterstained with 1 µg/ml 4′,6-diamidino-2-phenylindole (DAPI, Sigma D9542) in PBS for 10 min at RT. Inserts were carefully mounted with Mowiol^®^ 4–88 (Sigma, 81,381) and covered with a glass coverslips. A confocal laser scanning microscope (Zeiss microscopes, Jena, Germany) with a C-Apochromat 40 × /1.2 W Corr M27 objective was used to obtain z-stack images. The confocal pinhole was set to 1 AU to optimise the z-sectioning in the confocal mode.

### Alkaline phosphatase (ALP) activity assay

For ALP activity assays, advanced intestinal co-cultures were treated with 50 µg/ml SAS for 48 h in 6-well inserts. Aspirin (10 µM) and trifluoperazine (100 µM) were explored as potential positive controls. After the treatments, the inserts were washed with ice-cold PBS and 600 µl/insert lysis buffer was added to the apical side (Cosín-Roger et al. 2013). The inserts were stored until further processing at − 20 °C. To investigate the activity of the alkaline phosphatase the plates were thawed for 15 min at 600 rpm on a shaker plate (Heidolph Titramax 101). The cell layer was detached with the help of a cell scraper and homogenised for 2 min in a sonification bath (Bandelin Sonorex Super RK 156 BH). The homogenate was centrifuged at 16,366 g at 4 °C for 30 min. A p-nitrophenol (pNP; Sigma, 1048) standard curve was prepared with 0 and 4, 8, 12, 16, 20, 30, 50 nmol/well. The supernatants of the homogenates were diluted 1:40 in diethanolamine buffer and 80 µl of this mixture was added to the wells of a 96-well plate. Additionally 50 µl of 5 mM p-nitrophenylphosphate (pNPP; Sigma, 71,768) was added to all sample wells. The reaction was incubated for 30 min in the dark and stopped with 20 µl 3 M NaOH. Absorbance was measured with a multi-well plate reader (Mithras^2^ LB943, Berthold Technologies) at 405 nm. The total protein was determined with the Pierce™ BCA Protein Assay Kit (Thermo Fischer, 23,225) according to the manufacturer’s protocol. Interference analysis was performed at three different concentrations of SAS (0.05, 3.13 and 50 µg/ml) in one independent experiment with two technical replicates. None of the SAS quenched the pNP signal, reduced pNPP to pNP or exhibited auto-absorption up to a concentration of 50 µg/ml (data not shown).

### Lipid uptake

To assess potential effects of SAS on lipid uptake, advanced intestinal co-cultures in 12-well inserts were treated with 50 µg/ml SAS for 48 h. For the positive control investigations, 22 d differentiated co-cultures were treated with 50 µg/ml of the fatty acid synthase inhibitor C75 for 24 h (Accioly et al. 2008). After the treatment the medium was removed and the cultures were washed with PBS. Inserts were incubated for 10 min with 250 µl of 20 µM BODIPY™ 500/510 C1, C12 (Thermo Fischer, D3823) in 0.1% BSA in PBS according to the manufacturer’s protocol. Then inserts were washed with 500 µl of ice cold 0.1% BSA in PBS and complete DMEM made from phenol red free medium was added to the apical (590 µl) and basal (1.5 ml) compartment. The cells were incubated for 1 h at standard growth conditions and the fluorescence was measured with a multi-well plate reader at 485/528 nm (excitation/emission).

### Iron uptake

72 h before the treatment of advanced intestinal co-cultures in 12-well inserts with SAS, 1 mg/ml SAS was pre-incubated in 5% BSA under standard growth conditions to establish a protein corona on the particles. Then the co-cultures were treated for 48 h with 50 µg/ml BSA-pre-coated SAS in iron free medium (phenol red free DMEM (Gibco) supplemented with 10 mM PIPES, 11 µM hydrocortison, 50 µg/ml insulin, 0.02 µM sodium selenite, 0.05 µM triiodothyronine, 0.2 µg/ml EGF and 1% (v/v) PSN (all from Sigma besides EGF which was ordered from Thermo Fischer)) (Christides et al. 2018; Perfecto et al. 2018). All inserts besides the background controls additionally received 30 µM ferric ammonium citrate after the 48 h treatment with SAS. 2.5 mM CaCl_2_ or 600 µM vitamin C in iron free medium were used as positive controls (Perfecto et al. 2018) and were added to untreated co-cultures during the final 24 h. After incubation for another 24 h at standard growth conditions the inserts were washed with PBS, lysed with 200 µl Cell Lytic M buffer (Sigma, C2978) and centrifuged at 14,000 g for 15 min at 4 °C. The ferritin amount was measured with human Ferritin ELISA (Sigma, RAB0197) according to the manufacturer’s protocol. In brief, the supernatant was diluted 1:2 in sample buffer and 100 µl/well were added per well of a pre-coated 96-well plate. After a 2.5-h incubation at RT the provided plate was washed and incubated for 1 h with detection antibody. The HRP-Streptavidin antibody was added for additional 45 min. After 30 min of incubation with the ELISA Colorimetric TMB Reagent the reaction was stopped with the stop solution and absorbance was immediately measured in a multi-well plate reader (Mithras^2^, Berthold Technologies) at 450 nm. Interference analysis was performed at 50 µg/ml of SAS in one independent experiment with 2 technical replicates. None of the SAS showed intrinsic catalytic activity, influenced the optical density or bound to the antibodies or the antigen up to a concentration of 50 µg/ml (data not shown).

### Detection of cytokine release by ELISA

After 24 h exposure to 50 µg/ml SAS, 200 µg/ml PS-amine or 10 ng/ml IL-1β, the release of IL-8 and CCL2 was quantified in cell-free supernatants by ELISA. The cytokine amount was measured with human IL-8 ELISA (Invitrogen, 88–8086) and CCL2 ELISA (Invitrogen, 88–7399) according to the manufacturer’s protocol. In brief, the supernatant was diluted 1:5 (IL-8) or 1:2 (CCL2) in phenol red free complete medium and 100 µl/well were added. After a 2-h incubation at RT the plate was washed and incubated for 1 h with detection antibody. The avidin-HPR antibody was added for additional 30 min and then the reaction was developed with the substrate solution for another 15 min before it was stopped with 2 N H_2_SO_4_. Absorbance was measured immediately in a multi-well plate reader (Mithras^2^, Berthold Technologies) at 450 nm. Interference analysis was performed at 3.13 and 50 µg/ml of SAS in one independent experiment with two technical replicates. None of the SAS showed intrinsic catalytic activity, influenced the optical density or bound to the antibodies or the antigens up to a concentration of 50 µg/ml (data not shown).

### Statistics

Results are presented as mean ± standard deviations of three independent experiments, which were run with at least two technical replicates each. Statistical analysis was conducted with GraphPad Prism 8 software using a one-way analysis of variance (ANOVA; 95% confidence interval) followed by the Dunnett’s or Bonferroni’s multiple comparisons test or Kruskal Wallis with Dunn’s multiple comparisons test for the evaluation of the qPCR results.

## Results

### SAS did not induce acute cytotoxicity in advanced intestinal co-cultures

**Fig. 2:**
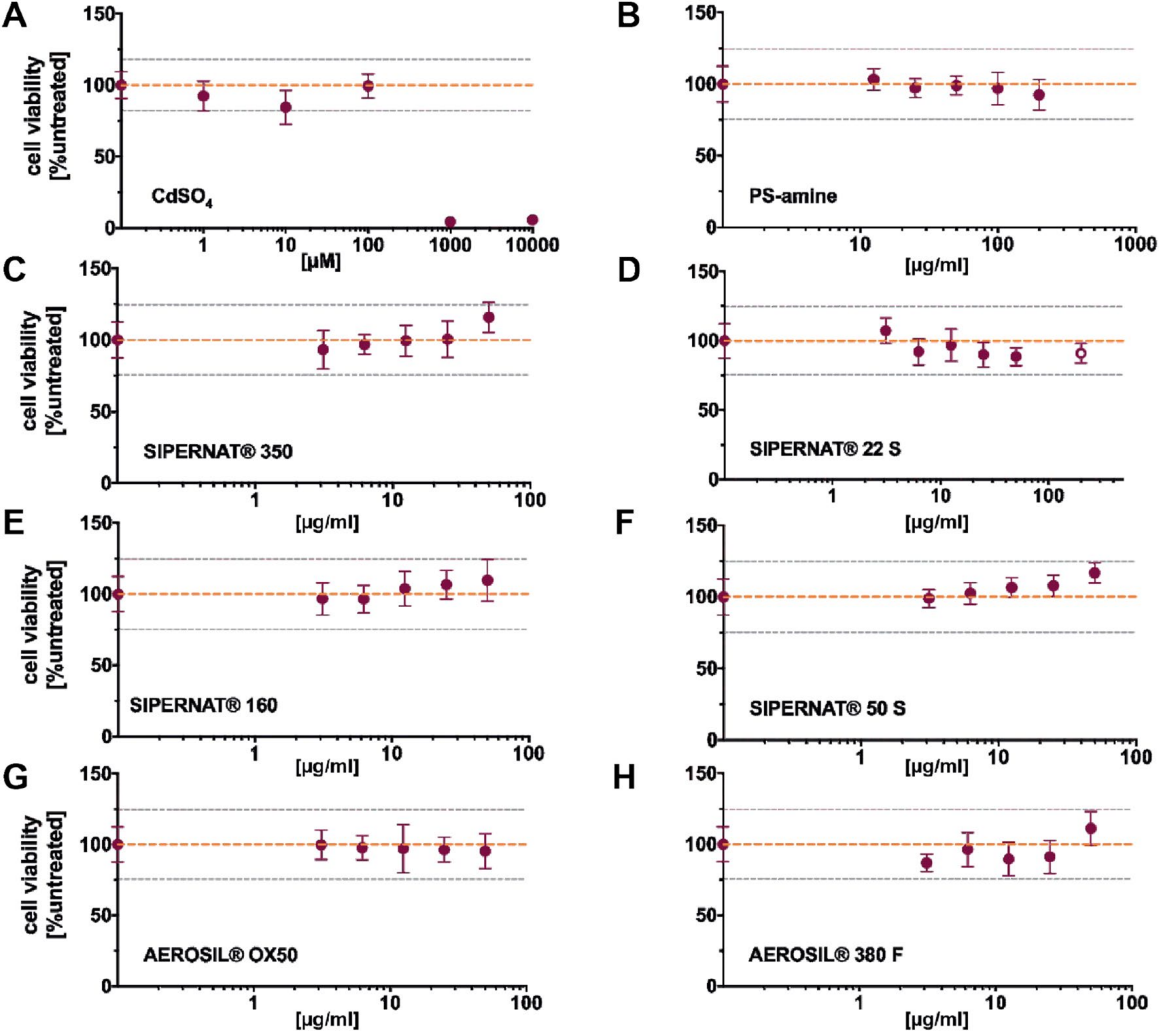
Impact of SAS on cell viability of advanced intestinal co-cultures after 48 h of exposure. Following incubation of the advanced co-culture model with various concentrations of different SAS for 48 h cell viability was assessed with the MTT assay. **a** CdSO_4_ served as a chemical positive control. **b** PS-amine was used as a particle positive control. **c** SIPERNAT^®^ 350. **d** SIPERNAT^®^ 22 S. Here also a higher concentration of 200 µg/ml (hollow symbol) was applied. **e** SIPERNAT^®^ 160. **f** SIPERNAT^®^ 50 S **g** AEROSIL^®^ OX50. **h** AEROSIL^®^ 380 F. Mean values and corresponding standard deviations from three independent experiments with four technical replicates each are shown. The orange dashed line and the grey dotted lines resemble the mean value of the solvent control sample and twice the corresponding standard deviations, respectively. ***P ≤ 0.001, ****P ≤ 0.0001 compared to the solvent control samples (colour figure online)

To assess the influence of SAS materials on the intestinal barrier in vitro, an advanced human intestinal co-culture model based on Caco-2/HT-29/Raji B cells (Fig. 1) was established and carefully characterized (Fig S1). The viability of the advanced intestinal co-cultures was determined after 24 h and 48 h of treatment with SAS using the MTT assay. All six SAS materials did not induce any decrease in cell viability below 86% after 48 h (Fig. 2 and S2). Four of the SAS materials (SIPERNAT^®^ 350, SIPERNAT^®^ 160, SIPERNAT^®^ 50 S and AEROSIL^®^ 380) even demonstrated a slight concentration-dependent increase in cell viability after 48 h of treatment (Fig. 2). For SIPERNAT^®^ 22 S, a higher concentration of 200 µg/ml was included since we previously observed a significant decrease in the viability of differentiated Caco-2 cultures to 83% after 48 h exposure to 50 µg/ml (Hempt et al. 2020). Even at this high concentration, SIPERNAT^®^ 22 S did not considerably decrease cell viability (91 ± 6.7%) in the advanced co-cultures after 48 h. In contrast, CdSO_4_ significantly reduced cell viability in the co-cultures in a dose-dependent manner after 24 h and 48 h of exposure (Fig. 2 and S2). PS-amine particles were chosen due to previous reports that cationic amine-functionalised PS-particles are linked to cell death in other cell types (Hempt et al. 2020; Xia et al. 2008, 2006). However, in the advanced co-cultures containing a mucus barrier, no significant decrease in cell viability was detected up to a concentration of 200 µg/ml and 48 h of treatment with PS-amine.

### SAS did not influence functional endpoints in advanced intestinal co-cultures besides iron uptake for two SAS materials

#### Barrier integrity

**Fig. 3:**
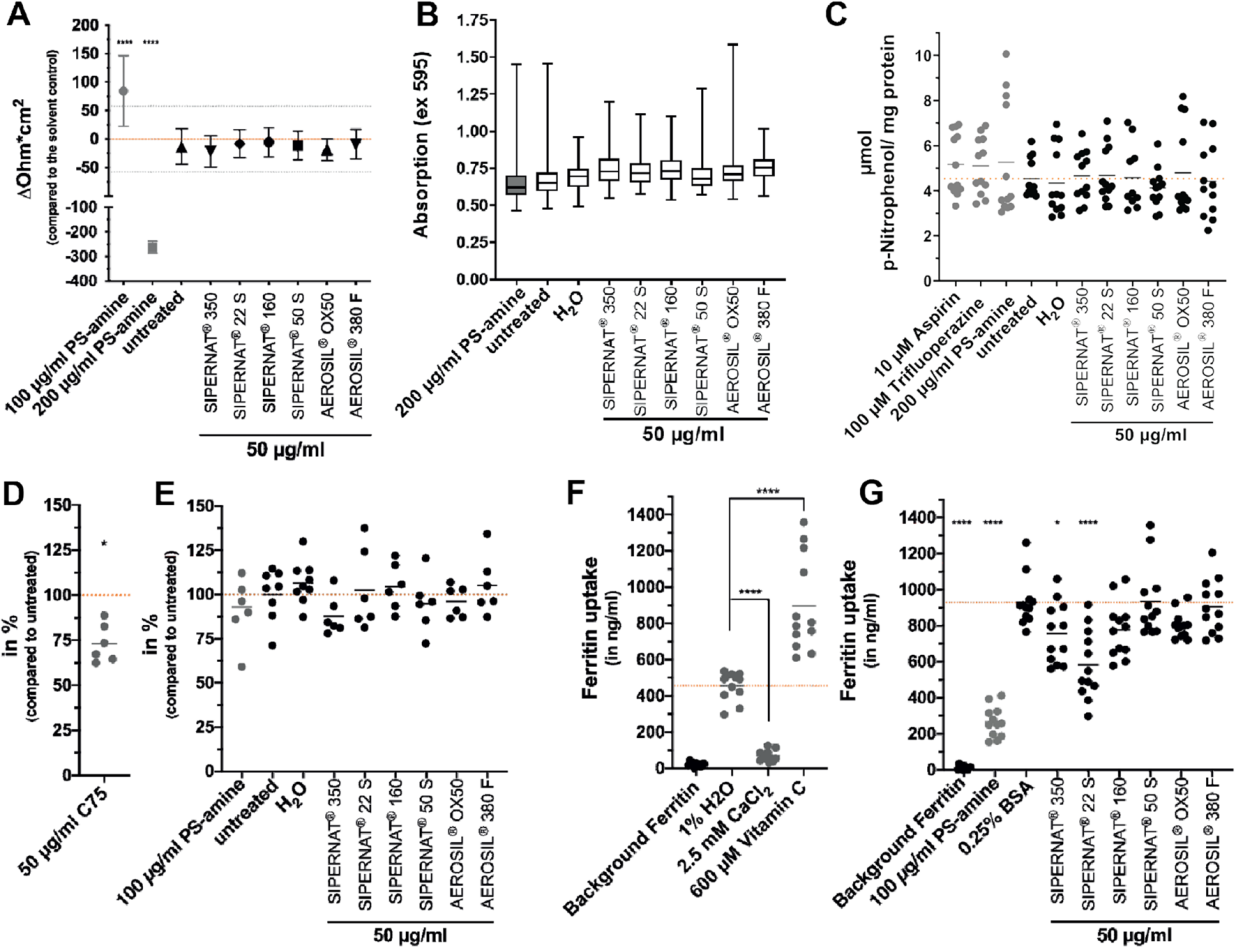
Impact of SAS on physiological functions of the advanced intestinal co-cultures after 48-h exposure including intestinal barrier integrity, mucus coverage, microvilli function as well as lipid and iron uptake. **a** After 48 h of treatment with 50 µg/ml SAS and 200 µg/ml PS-amine TEER values were determined. Mean values and corresponding standard deviations for the SAS materials from six independent experiments and for PS-amine from three independent experiments with two technical replicates each are shown. The dashed orange lines and the grey dotted lines resemble the mean value of the solvent control sample and twice the corresponding standard deviations, respectively. **b** After incubation of the advanced co-culture with 50 µg/ml of the indicated SAS the mucus coverage was analysed by staining inserts with alcian blue and measuring absorption for 25 areas (5 × 5 µm each) per insert. Median, 25th and 75th percentile values from three independent experiments with two technical replicates each are shown in the box-whiskers plots. **c** The activity of alkaline phosphatase (ALP) was assessed after the treatment of co-cultures with 50 µg/ml SAS for 48 h by measuring the conversion of pNPP to pNP. 10 µM aspirin, 100 µM trifluoperazine and 200 µ/ml PS-amine were included as potential positive controls. Data points from three independent experiments performed in duplicates and measured in two technical replicates each are shown. Mean values of all data points are shown as horizontal lines. **d** The fatty acid synthase inhibitor C75 added at a concentration of 50 µg/ml for 24 h served as a chemical positive control for the investigation of lipid uptake. **e** After 48-h incubation of the advanced co-culture with the indicated SAS the lipid uptake was determined. Data points from three independent experiments performed in duplicates and measured in two technical replicates each are shown. Mean values of all data points are shown as horizontal lines. (D and E). **f** After a 24-h incubation of the advanced co-culture with 2.5 mM CaCl_2_ (iron uptake reducer) or 600 µM vitamin C (iron uptake enhancer) the ferritin content in the cells was analysed. **g** After treatment with 50 µg/ml SAS for 48 h the amount of ferritin in the cells was measured. Data points from three independent experiments performed in duplicates and measured in two technical replicates each are shown. Mean values of all data points are shown as horizontal lines. (F and G). The dashed orange lines represent the mean values of solvent control samples (A, F, G) or untreated control samples (C, E). *P ≤ 0.05, ****P ≤ 0.0001 compared to the solvent controls or untreated control samples (colour figure online)

To examine the potential effect of the SAS materials on the barrier integrity of advanced intestinal co-cultures, TEER values were determined after 48 h of exposure to 50 µg/ml SAS. None of the six SAS materials induced significant changes in the TEER values compared to the solvent control (Fig. 3a). In contrast, 200 µg/ml PS-amine particles diminished TEER values by − 263.4 ± 22.6 Ω*cm^2^ as compared to solvent control H_2_O.

#### Mucus production and coverage

The effect of SAS on mucus coverage of advanced intestinal co-cultures was evaluated by alcian blue staining and absorption measurements. No disruption of the mucus layer was apparent from visual inspection after treatment with 50 µg/ml SAS for 48 h but slight differences in the intensity of the stainings across the inserts made a comparison difficult. For a quantitative readout, we, therefore, measured the optical absorbance of 25 areas per each insert in a plate reader, which showed a broad distribution in the staining intensities. Nevertheless, median values in alcian blue staining were similar for the SAS treated cultures and the solvent control (Fig. 3b). Furthermore, we also assessed the impact of SAS on mucin production. *MUC1* is one of the most studied membrane-associated mucins and is upregulated in the inflamed intestine (McAuley et al. 2007) and in ulcerative colitis in humans (Longman et al. 2006). The expression of the *MUC1* gene was significantly downregulated after treatment with 200 µg/ml PS-amine for 24 h (Fig S3A). For the SAS materials, a decrease in *MUC1* expression was only observed after treatment with 50 µg/ml SIPERNAT^®^ 160 (Fig S3A). However, these effects were small with mean fold changes compared to the solvent control of − 1.72 ± 1.56 for PS amine and − 1.30 ± 0.44 for SIPERNAT^®^ 160.

#### Alkaline phosphatase activity

Alkaline phosphatase is present in the brush border of enterocytes and its activity has been exploited as a marker to confirm microvilli function and potential disruption of the microvilli layer (Fan et al. 1999; Miura et al. 1982). ALP activity was assessed after 48 h of exposure to 50 µg/ml SAS by measuring the turnover of p-nitrophenylphosphate to p-nitrophenol by alkaline phosphatase. None of the six SAS materials induced any significant changes in the alkaline phosphatase activity compared to the solvent control water or the untreated control (Fig. 3c). The values for all treatment varied considerably. The ALP activity assay has already been applied in previous studies, however, without including any positive controls in the Caco-2/HT-29 co-culture (Guo et al. 2017, 2018). Here, we explored the non-steroidal anti-inflammatory drug (NSAID) aspirin (10 µM) and trifluoperazine (100 µM) as potential controls since NSAID have been described to induce the ALP activity in rats (Sood et al. 2008) while trifluoperazine has been shown to decrease ALP activity in the rat intestine (Wang and Gilles-Baillien 1992). Both, aspirin and trifluoperazine did not affect ALP activity in the advanced co-culture and a suitable positive control remains to be identified. Gene expression levels of the *ALP* gene were below the detection limit of the qPCR and therefore could not be analysed.

#### Lipid uptake

The uptake of lipid components at the brush border of the enterocytes is an essential physiological function of the small intestine (Campbell et al. 2019). A lipid uptake assay, that is exploiting the fluorescent fatty acid analog BODIPY™ 500/510 C1, C12 as a traceable model lipid, was performed to assess a potential impact of SAS on lipid uptake in advanced intestinal co-cultures. The fatty acid synthase inhibitor C75 has been described as a positive control in Caco-2 monocultures (Accioly et al. 2008). Treatment of advanced co-cultures with 50 µg/ml C75 for 24 h significantly reduced lipid uptake, confirming C75 as a positive control, also in mucus-expressing co-cultures (Fig. 3d). In contrast, the six SAS materials as well as PS-amine did not induce any significant differences in the uptake of the fluorescent lipid after 48 h (Fig. 3e).

To further confirm the absence of adverse effects on lipid uptake, we measured the gene expression levels of *GPR120,* a receptor for omega 3 fatty acids that is involved in anti-inflammatory effects (Oh et al. 2010). *GPR120* expression was significant increased after 24 h of treatment with 50 µg/ml SIPERNAT^®^ 350 but downregulated for the untreated control compared to the solvent control (Fig S3B). The treatment with fatty acid synthase inhibitor C75 and the other SAS did not result in a gene expression change of the *GPR120* gene*.*

#### Iron uptake

Ferritin uptake is a key physiological function exhibited by enterocytes *in vivo* (Campbell et al. 2019)*.* The treatment with 2.5 mM calcium chloride, an iron uptake inhibitor (Perfecto et al. 2018), showed a significant decrease in ferritin uptake in advanced intestinal co-cultures (Fig. 3f). The iron uptake enhancer vitamin C (Perfecto et al. 2018) significantly increased ferritin uptake in advanced co-cultures (Fig. 3f). Similarly, 100 µg/ml PS-amine induced a significant reduction in ferritin uptake compared to the solvent control 0.25% BSA. The treatment with the six SAS materials showed that SIPERNAT® 350 and SIPERNAT_®_ 22 S reduce the ferritin uptake significantly in the advanced co-culture (Fig. 3g).

To investigate if the changes of ferritin uptake after the treatment with the two SAS were also detectable with another pathway, the gene expression of the *divalent metal transporter 1* (*DMT1*) was investigated. The divalent metal transporter *DMT1* has been previously investigated to identify shifts in intestinal iron absorption (Zoller et al. 2001). The treatment of advanced co-cultures with 50 µg/ml SAS materials did not result in a change in the gene expression of the *DMT1* gene in comparison to the solvent control (Fig S3C). Only for the treatment with 200 µg/ml PS-amine a significant reduction in *DMT1* gene expression was observed.

### SAS did not induce the release of the cytokines IL-8 and CCL2 in advanced intestinal co-cultures

**Fig. 4:**
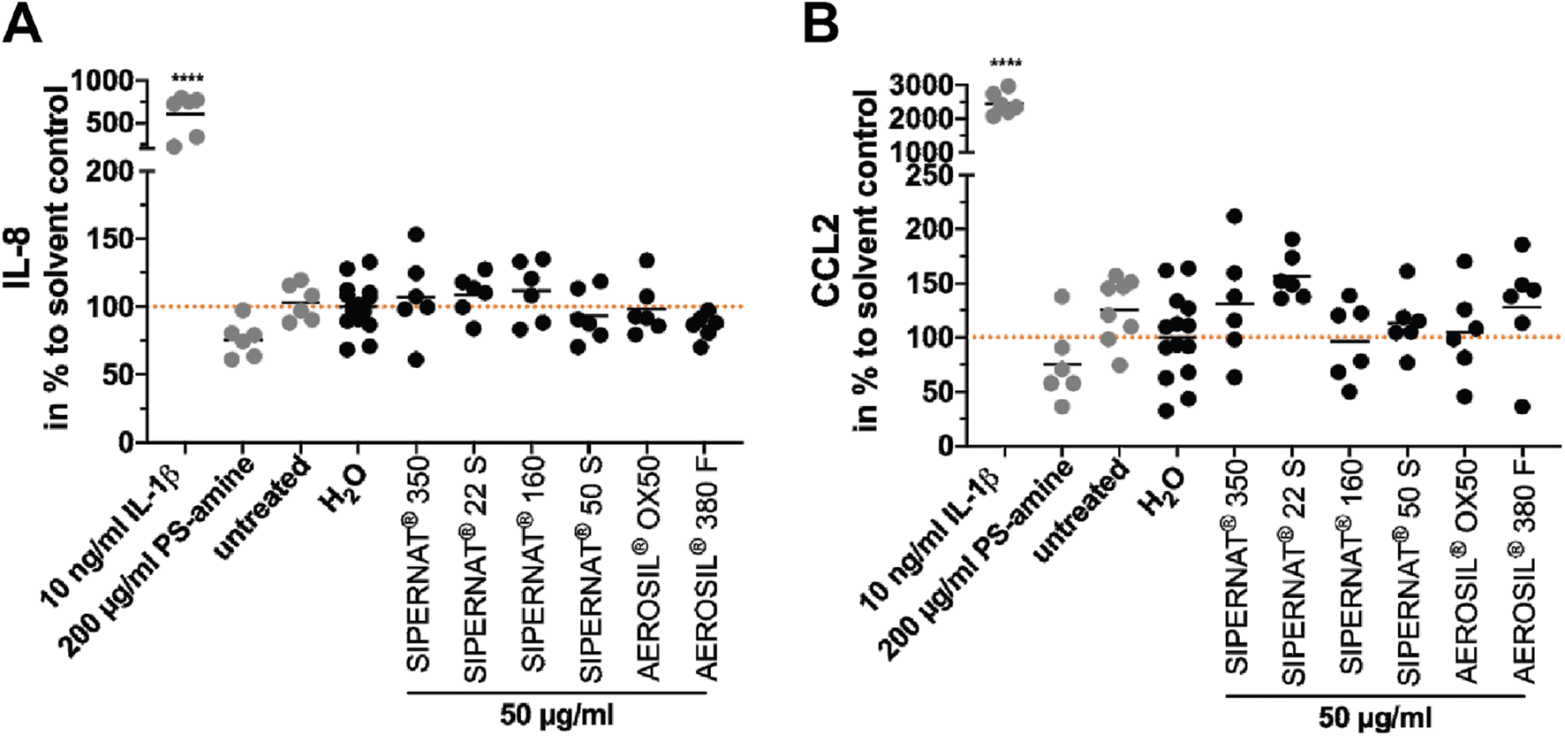
Impact of SAS on the release of the inflammatory cytokines IL-8 and CCL2 in advanced intestinal co-cultures after 24 h of exposure. After 24 h incubation of the advanced co-culture with the indicated SAS the cytokine of IL-8 (**a**) and CCL2 (**b**) release was determined. The cytokine IL-1β at a concentration of 10 µg/ml for 24 h served as a chemical positive control. Data points from three independent experiments with two technical replicates each are shown. Mean values of all data points are shown as horizontal lines. The dashed orange lines resemble the mean value of the solvent control sample. *P ≤ 0.05, ****P ≤ 0.0001 compared to the solvent control (colour figure online)

The release of the chemotactic and pro-inflammatory cytokines IL-8 and CCL2 was detected in the apical compartment after 24 h of treatment with the different SAS materials. None of the six SAS materials affected the release of the inflammatory cytokines IL-8 and CCL2 compared to the solvent control H_2_O (Fig. 4). The treatment with the positive control IL-1β resulted in a significant increase in the secretion of both cytokines (Fig. 4). Only after exposure of advanced co-cultures with 200 µg/ml PS-amine, a slight tendency for decreased CCL2 levels was observed, which was however not statistically significant.

Similarly, gene expression levels of *IL-8* were not altered after the treatment with the SAS materials for 24 h (Fig S3D). Stimulation with 10 ng/ml IL-1β resulted in a significant increase in the gene expression of *IL-8*. Collectively, these data indicate that SAS do not have an acute effect on pro-inflammatory cytokine expression and secretion in advance intestinal co-cultures.

## Discussion

Advanced intestinal co-culture models are more physiologically relevant than monoculture systems and were thus suggested to be superior for the safety assessment of ingested (nano)materials (García-Rodríguez et al. 2018b; Lehner et al. 2020; Saez-Tenorio et al. 2019; Sohal et al. 2020; Ude et al. 2017, 2019; Vila et al. 2018). Here, we applied a mucus-secreting intestinal co-culture model composed of Caco-2, HT-29 and Raji B cells as first reported by Schimpel et al. (2014). The mucus layer has previously been shown to constitute an essential physical–chemical barrier for many nanomaterials (Huck et al. 2019; Liu et al. 2014; Schneider et al. 2017) and should, therefore, be included in the evaluation of transport and effect studies of ingested food additives at the intestinal barrier. However, the presence of a mucus layer can be problematic for some conventional cytotoxicity assays due to limited penetration of reagents from or to the cells. For instance, we were not able to apply the resazurin viability assay to co-cultures most likely due to inefficient penetration and release of the resazurin/resorufin across the mucus layer (own unpublished data). Moreover, some positive assay controls that worked in the Caco-2 monocultures showed less pronounced effects (e.g., C75 or PS-amine) or did not work at all (aspirin or trifluoperazine) in the co-cultures (Hempt et al. 2020 and own unpublished data). Nevertheless, the following assays were compatible with mucus-secreting intestinal co-cultures including a positive chemical or particle control: MTT assay, TEER measurements, lipid and iron uptake assays as well as cytokine ELISA. For mucus coverage (alcian blue staining) and ALP activity, we did not yet succeed to identify a suitable positive control.

Besides a physiologically relevant biological model, it is important to use realistic in vitro dose ranges comparable to *in vivo* human exposures by integrating available human uptake data, knowledge of GIT physiology and computational in vitro dosimetry models (Sohal et al. 2018). For food-relevant SAS, estimated exposure doses for daily intake are 2.41 µg/cm^2^ (Sohal et al. 2020) or 0.02–11 µg/cm^2^ (Hempt et al. 2020). According to our previous study where we modelled the delivered dose for all the SAS materials (Hempt et al. 2020), the applied doses of 50 µg/ml correspond to deposited doses of 11.8 µg/cm^2^ for SIPERNAT^®^ 350, 10.3 µg/cm^2^ for SIPERNAT^®^ 22 S, 7.6 µg/cm^2^ for SIPERNAT^®^ 160, 10 µg/cm^2^ for SIPERNAT^®^ 50 S, 6.1 µg/cm^2^ for AEROSIL^®^ OX50 and 1.5 µg/cm^2^ for AEROSIL^®^ 380 F (Hempt et al. 2020), which are in a realistic exposure dose range.

In our study, all investigated SAS materials did not affect cell viability/metabolic activity of the intestinal co-cultures after exposure up to 50 µg/ml for 48 h. Similarly, the particle control PS-amine, which induced a slight cytotoxic response in differentiated Caco-2 monocultures (Hempt et al. 2020), did not decrease cell viability in the co-cultures, presumably due to the presence of a protective mucus layer. A decreased sensitivity in regards of cell viability and barrier integrity has previously been reported for other nanomaterials including silver, SiO_2_ and CuO nanoparticles when comparing the results of a Caco-2 monoculture with an intestinal co-culture model (Cornu et al. 2020; Saez-Tenorio et al. 2019; Ude et al. 2019, 2017; Vila et al. 2018). However, a recent study found a considerable dose-dependent decrease in the viability/metabolic activity (PrestoBlue™ viability assay) of intestinal co-cultures treated with a food-grade SAS material (AEROSIL^®^ 200 F) compared to the Caco-2 monoculture (Sohal et al. 2020). The different outcome compared to our study could be due to slight differences in the experimental setup or characteristics of the applied SAS materials. For instance, AEROSIL^®^ 200 F displayed a very slow settling rate (only 0.3% of the administered dose was delivered to the cells after 24 h) (Sohal et al. 2020) whereas deposition was much higher for all of the six SAS materials investigated in this study (deposited fractions between 5 and 63%) (Hempt et al. 2020).

Similar to the absence of adverse effects of SAS on cell viability, we did not observe any impact of the investigated SAS on barrier integrity. However, biological effects became apparent in some of the intestine-specific functional assays for selected SAS materials. For iron uptake, two of the SAS materials (SIPERNAT^®^ 350 and SIPERNAT^®^ 22 S) significantly reduced the uptake of ferritin after 48 h treatment with 50 µg/ml of the materials. However, this was not associated with a change in the expression of the *divalent metal transporter 1 (DMT1)*, in contrast to the particle control PS-amine, which reduced both, ferritin uptake and *DMT1* expression. Adverse effects were also reported for TiO_2_ and SiO_2_ nanoparticles, which induced a significant decrease in iron transport but no change in iron uptake after 5 d chronic exposure in Caco-2/HT-29 co-cultures (Guo et al. 2018, 2017). Therefore, iron uptake and/or translocation may be prone to disturbance from different types of materials; however, there is no clear pattern as to which material properties could be responsible for these effects. For the SAS, the two types of materials that significantly reduced ferritin uptake had a relatively low specific surface area and were produced by precipitation, but two other SAS with equally low specific surface area (SIPERNAT^®^ 160 and AEROXIL^®^ OX50) only showed a tendency for reduced ferritin uptake. It remains to be investigated whether the moderate effects of some SAS on ferritin uptake persist or if the cells develop adaptive responses.

Uptake of lipids was not compromised by any of the investigated SAS materials in the intestinal co-cultures after treatment with 50 µg/ml for 48 h. This is in line with a previous study, which described that 30 nm SiO_2_ nanoparticles reduced the uptake of lipids only after long-term exposure (5 d) but not acute exposure (4 h) in Caco-2/HT-29 co-cultures (Guo et al. 2018). We further studied potential effects of SAS on the expression of the receptor for middle and long-schain fatty acids *GPR120*. This receptor was slightly upregulated only after exposure to SIPERNAT^®^ 350, which is a precipitated form of SAS with the lowest specific surface area of all investigated precipitated SAS materials. *GPR120* is known to influence glucose uptake and inflammation (Oh et al. 2010; Song et al. 2017; Zhang and Leung, 2014). However, we did not detect any significant changes in the release of the pro-inflammatory cytokines IL-8 and CCL2 after treatment with SIPERNAT^®^ 350 or any other SAS material. Nevertheless, future studies are warranted to understand the effects of prolonged exposure to SIPERNAT^®^ 350 on lipid uptake.

Assessment of mucus coverage and production did not reveal adverse effects for most SAS despite a slight decrease of *MUC1* expression after 24 h of exposure to 50 µg/ml SIPERNAT^®^ 160. *MUC1* expression was also reduced by PS-amine but in both cases, this did not change the overall mucus coverage of the co-cultures even after 48 h of treatment. It is possible that the effect on *MUC1* expression was only transient as previously described for Caco-2/HT-29 co-cultures treated with silver nanoparticles (Saez-Tenorio et al. 2019) or that other mucins present in the mucus layer could compensate for this decrease. Further studies are needed to understand if subtle effects on the mucus barrier might result in long-term consequences for intestinal barrier function.

Finally, none of the SAS materials affected the activity of ALP, suggesting that they do not affect microvilli layer integrity. However, since a suitable positive control is still lacking, it is unclear how sensitive this assay is to detect microvilli disruptions. In other studies, a significant increase in ALP activity has been detected for acute (4 h) and chronic (5 d) exposure of Caco-2/HT-29 co-cultures with TiO_2_ nanoparticles (Guo et al. 2017) or for chronic exposures with SiO_2_ nanoparticles (Guo et al. 2018). However, the latter work used non-food grade 20–30 nm SiO_2_ nanoparticles, which have highly distinct properties from the here studied SAS materials.

**Fig 5:**
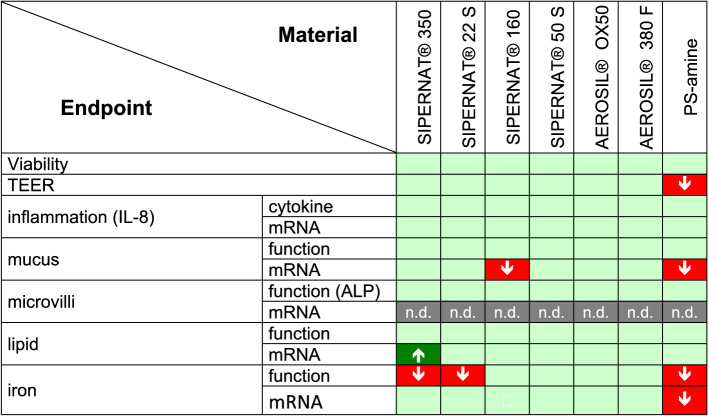
Summary of the effects of the different SAS and PS-amine on advanced intestinal co-cultures. Shown are effects on different endpoints on functional (function) or gene expression level (mRNA) with: significant increase (green), in the range of the solvent control (light green) and significant decrease (red). n.d. = not detectable (grey) (colour figure online)

The six SAS material investigated in this study have been chosen to represent different production routes (wet and thermal), specific surface area (low, middle, large) and total silanol content (low, high). Pairs of SAS materials that vary in only one property are represented in this selection to investigate potential structure–activity relationships (SAR). A table summarising the biological effects of the different SAS has been compiled (Fig. 5) to aid the identification of potential SAR.

One hypothesis of a SAR for SAS has been previously put forward by Zhang et al. (2012), who suggested that toxicity to bronchial epithelial (BEAS-2B) and macrophage (RAW 264.7) cells observed for fumed but not colloidal SAS was due to the presence of siloxane rings in fumed SAS. More recently, Rubio et al. (2019) confirmed that surface silanol content plays an important but not exclusive role in cellular toxicity and surface reactivity. They found that amorphous silica nanoparticles with lower total silanol content exhibited larger adverse cellular effects in RAW cells while normal small airway epithelial cells (SAEC) did not show any sign of toxicity (Rubio et al. 2019). In general, precipitated SAS materials have a higher total silanol content than pyrogenic (fumed) SAS (Fruijtier-Pölloth 2012; Zhang et al. 2012), which we also observed for the food relevant SAS used in this study. Nevertheless, differences in total silanol content did not correlate with the few observed biological responses in advanced intestinal co-cultures, probably due to limited penetration of the particles across the mucus layer. It has been recently shown that only a small fraction of the administered E 551 did cross a Caco-2/Raji co-culture in vitro (Yu et al. 2020) even in the absence of a mucus layer. Therefore, our findings further support a cell type-specific toxicity of SAS in dependence of total silanol content.

Regarding specific surface area of the SAS, we did not identify a clear correlation between this structural parameter and the observed biological responses in the intestinal co-cultures. SIPERNAT^®^ 350, with the lowest specific surface area showed some effects on iron uptake and expression of the receptor for middle and long chain fatty acids *GPR120*. However, AEROSIL^®^ OX50, which was produced by a different production route (thermal) but has a similarly low specific surface area, did not induce any acute toxic response in intestinal co-cultures. It is therefore possible that not a single characteristic but a combination of material properties is determining the biological activity, e.g. that only SAS with a small specific surface area and produced by a wet process interfere with iron uptake in intestinal cells. Due to the limited biological responses induced by the investigated SAS, it was not possible to identify further SAR for SAS in intestinal co-cultures.

Overall, we did not identify a single characteristic or a specific combination of properties that would be highly critical in regard to intestinal barrier impairment in vitro, and most of the studied SAS did not induce any adverse effects on a broad variety of functional endpoints.

## Conclusion

Six food-relevant SAS materials were assessed for their short-term impact on the human intestinal barrier in vitro at concentrations relevant to dietary exposure. Despite considerable differences in specific surface area or silanol content, the investigated SAS did not affect cell viability, barrier integrity, inflammatory responses or microvilli function. These results are highly relevant for the hazard characterisation of E 551 indicating a low risk for a negative biological response at the intact intestine from a short-term single exposure to food-relevant SAS, independent of the applied production route and the resulting variations in particle characteristics. Only slight effects regarding expression of *MUC1* and *GPR120* as well as on iron uptake were observed for few SAS. Future studies including prolonged repeated exposures should address whether the induced alterations persist and could have a lasting impact on the integrity and function of the human intestinal barrier.

Previously formulated SAR between total silanol content and toxicity in monocytes was not confirmed for mucus-secreting co-cultures in this study, indicating cell type specific responses probably due to the presence of a protective mucus layer and/or the absence of monocytes in the cultures. The mucus barrier might also have resulted in a reduced cytotoxicity of the particle control PS-amine in the advanced co-culture model as compared to Caco-2 monocultures (Hempt et al. 2020). These observations highlight that the role of the mucus layer on nanomaterial interactions with the intestinal barrier should be investigated in more detail, and that mucus-secreting co-cultures are indispensable for a more realistic toxicity assessment of ingested materials. In addition, our study corroborates that inclusion of additional intestine-specific functional endpoints should be a central part of any safety assessment of ingested materials at the intestinal barrier in vitro to cover the full range of potential adverse effects.

## Electronic supplementary material

Below is the link to the electronic supplementary material.

Supplementary file2 (PDF 9840 kb)

## Data Availability

Not applicable.
