# Supplementary material for: Investigating the effects of differently produced synthetic amorphous silica (E 551) on the integrity and functionality of the human intestinal barrier using an advanced in vitro co-culture model
Source: Arch Toxicol. 2020 Dec 15;95(3):837–52. doi: 10.1007/s00204-020-02957-2 (PMC7904742; doi:10.1007/s00204-020-02957-2)
Supplement: Supplementary file 1 — Supplementary file2 (PDF 9840 kb) [file 204_2020_2957_MOESM1_ESM.pdf]

Supplementary data

Archives of Toxicology

Investigating the effects of differently produced synthetic amorphous silica (E 551) on the integrity and functionality of the human intestinal barrier using an advanced *in vitro* co-culture model

Claudia Hempt[1,2], Cordula Hirsch[1], Yvette Hannig[1], Alexandra Rippl[1], Peter Wick[1] and Tina Buerki-Thurnherr[1]

[1]*Laboratory for Particles-Biology Interactions, Empa, Swiss Federal Laboratories for Materials Science and Technology, Lerchenfeldstrasse 5, 9014 St. Gallen, Switzerland*

[2]*Department of Health Sciences and Technology, ETH Zürich, Zürich, Switzerland*

Corresponding author: tina.buerki@empa.ch

Materials and Methods

Scanning electron microscopy (SEM)

Advanced intestinal co-cultures cultivated for 21 d in 12-well inserts were washed twice in pre-warmed PBS and fixed with modified Karnovsky fixation solution (4 g paraformaldehyde, 50 ml ddH_2O, 5 ml glutaraldehyde 50%, 45 ml PBS without glucose and pH 7.4) for 1 h at RT (Kucki et al. 2017). Inserts were washed in PBS and dehydrated by ascending ethanol series (50–100% ethanol) followed by 30 min treatment with hexamethyldisilazane (HMDS). The cells were dried overnight in a fume hood and transferred to SEM sample holders with conductive adhesive tapes on the next day. Samples were sputter-coated (Sputter Coater Leica EM ACE600) with gold-palladium (ratio Au/Pd: 80/20, 10 nm thickness). SEM analysis was performed with a Hitachi S-4800 SEM operating at 2 kV.

RNA extraction and gene expression analysis by qPCR

Total RNA was extracted from the 6-well inserts of advanced co-cultures, which have been exposed to 50 µg/ml SAS, 200 µg/ml PS-amine, 10 ng/ml IL-1β, 2.5 mM $CaCl_2$, 50 µg/ml C75 or H_2O as the solvent control for 24 h, using RNeasy® Mini (QIAGEN, 74104) according to the manufacturer's protocol. DNase digestion has been performed either by RNase-Free DNase Set (QIAGEN, 79254) or DNA-free™ DNA Removal Kit (Invitrogen, AM1906) to discard residual DNA contamination. The iScript™ cDNA Synthesis Kit (BIORAD, 1708891) was used to obtain cDNA from 1000 ng of total RNA. The resulting cDNA was subjected to real-time PCR analysis on a CFX96 Dx (BIORAD). The relative expression of the brush border enzyme alkaline phosphatase (*ALP*), divalent metal transporter 1 (*DMT1*), mucin 1 (*MUC1*), a receptor for omega 3 fatty acids (*GPR120*) and *IL-8* was evaluated. Gene expression levels were normalised to the *housekeeping gene RLPL0*. The primer sequences are listed in the Table S1. Each reaction volume contained 50 ng cDNA, iQ™ SYBR® Green Supermix (BIORAD, 1708880), distilled H_2O and each primer pair was added at a final concentration of 200 nM. The cycling parameters were the following: an initial step of 95°C for 3 min, then 40 cycles of 95°C for 15 s, 60°C for 30 s and 72°C for 30 s. The qPCR data were analysed using the relative gene expression (ΔΔCT) method (Livak and Schmittgen, 2001).

Table S1: Primer sequence

	Forward primer (5'->3')	Reverse primer (5'->3')
ALP	GACAAGAAGCCCTTCACTGC	TGAGACCCCTGACCTTGACC
DMT 1	GACAGACGGTACCAACGGAA	ACTGGAAGCCAGAGTGAATGG
GPR120	GCCAGGACTGGTCATTGTGA	GCTGGCGTGAACTCTTAGGT
IL-8	GTCATTAGGTATCTGCCTTTTTGGT	GGACACTACTGGGAGTGACAAAG
MUC1	TTTCCAGCCCGGGATACCTA	TTCACGCCACTTCTCACCTC
RPLPO	CCTCGTGGAAGTGACATCGT	ATCTGCTTGGAGCCCACATT

Results

Characterisation of the advanced intestinal co-cultures

In vivo the small intestinal epithelium hosts enterocytes, goblet cells, Paneth cells, M-cells and in the underlying *Lamina propria,* macrophages, dendritic cells and T cells are present (Campbell et al., 2019). Altogether, these cells are important to execute the many physiological functions of the small intestine including digestion and absorption of nutrients, vitamin synthesis and the regulation of the fluid balance (Schneider and Feussner, 2017). To assess the influence of SAS materials on the intestinal epithelium *in vitro*, an advanced human intestinal co-culture model containing enterocytes (Caco-2), goblet cells (HT-29-MTX) and M cells (differentiated from Caco-2 cells in presence of Raji B-lymphocytes) with a continuous mucus layer was established (Fig 1). The differentiation status of the co-cultures was assessed by repeated TEER measurements over the time course of 21 days (Fig S1A). The TEER value gradually increased until day 14. After the addition of Raji B lymphocytes in the basolateral compartment the TEER values further increased until day 16, when the Raji B lymphocytes were removed from the basal compartment. In the period between 16-21 days of cultivation the TEER values reached a plateau of approximately 375 Ohm*cm^2 (Fig S1A). The steady increase of the TEER values indicates that intestinal co-cultures developed a tight epithelial barrier over the time course of 21 days. Only co-cultures that reached at least 350 Ohm*cm^2 were used for experiments. Similar TEER values are typically reported for *in vitro* co cultures containing Caco-2 and Raji cells (Antunes et al., 2013; Cabellos et al., 2017; des Rieux et al., 2007; Schimpel et al., 2014). Nevertheless,it should be noted that all these *in vitro* TEER values are significantly higher than physiological values reported for the intestine (e.g. 20 Ohm*cm^2 for the jejunum *in vivo*) (Hilgendorf et al., 2000).

Formation of a confluent barrier was further confirmed by CLSM micrographs showing the formation of close cell-cell contacts as visualised by staining for tight junction protein ZO-1 (Fig S1G-H). The presence of tight junctions between enterocytes (Assimakopoulos, 2011) is important for the tightness of the intestinal epithelium. At the fine structural level, SEM micrographs showed a homogeneous cell monolayer, including cells with or without microvilli structures on the apical surface (Fig S1B). Microvilli are a typical feature of intestinal epithelial cells (Shroyer and Kocoshis, 2011) and therefore confirm the polarisation of Caco-2 cells (Lea, 2015) similar to the *in vivo* situation. Moreover, microvilli were less abundant on HT-29 cells than on Caco-2 cells (Fig S1B), which is in agreement with *in vivo* findings reporting a similar difference in the brush border distribution between enterocytes and goblet cells (Shearman and Muir (1960). M-cells on the other hand have been described to not develop microvilli (Mabbott et al., 2013).

Mucus balls, a mucus artefact due to the fixation method, were also observed by SEM of intestinal co-cultures (Fig S1B). The formation of a confluent mucus layer covering the entire apical surface was verified by alcian blue staining (Fig S1D). Complete mucus coverage was only achieved for a seeding ratio of 75:25 Caco-2 to HT-29 cells while a seeding ratio of 9:1, which would more closely reflect the physiological ratio (Hilgendorf et al., 2000) resulted in an incomplete mucus layer (data not shown). Finally, immunostainings for cell type specific markers confirmed the presence of mucus producing goblet cells (MUC5AC positive) and M-cells (NKM 16-2-4 positive) (Fig S1E and F).

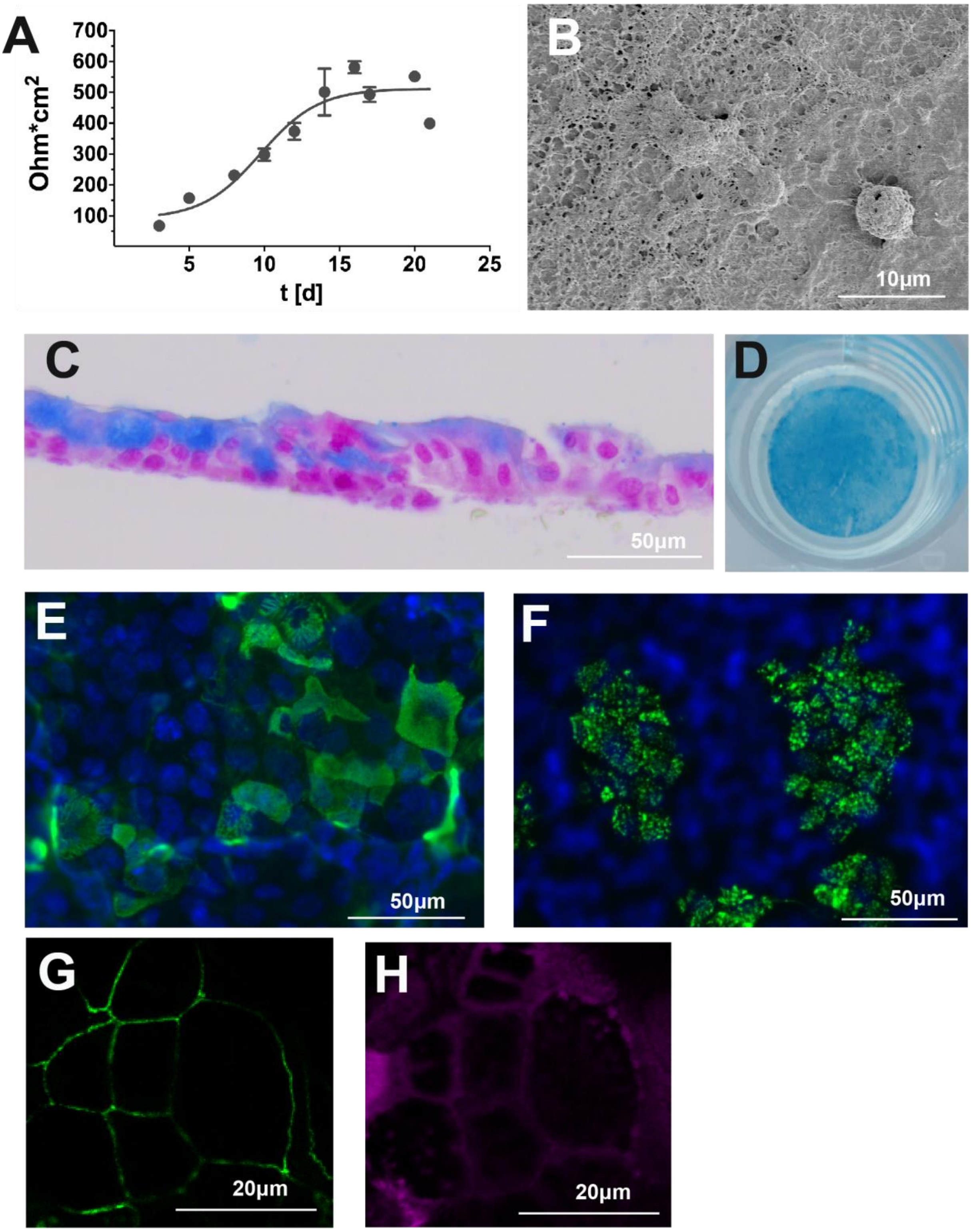

Figure S1: Verification of the advanced intestinal co-culture model

Characteristics of intestinal co-cultures were assessed after 21 d of cultivation. (A) TEER measurements over the time course of 21 days. (B) SEM micrograph of the apical surface of a representative advanced co-culture after 21 days of cultivation, showing a dense coverage with microvilli structures & mucus balls. (C) Alcian blue staining of a histological section. (D) Alcian blue staining of an untreated control sample (12 well Transwell® insert viewed from the top). (E-H) Representative confocal laser scanning microscopy image of the advanced co-culture after 21 days of differentiation . Cells were stained for the M-cell marker NKM 16-2-4 (green, E), the mucin MUC5AC (green, F), and nuclei (blue, E and F). (G and H) Staining of tight junction protein ZO-1 (green) and corresponding F-Actin (magenta). Representative images of two independent experiments are shown.

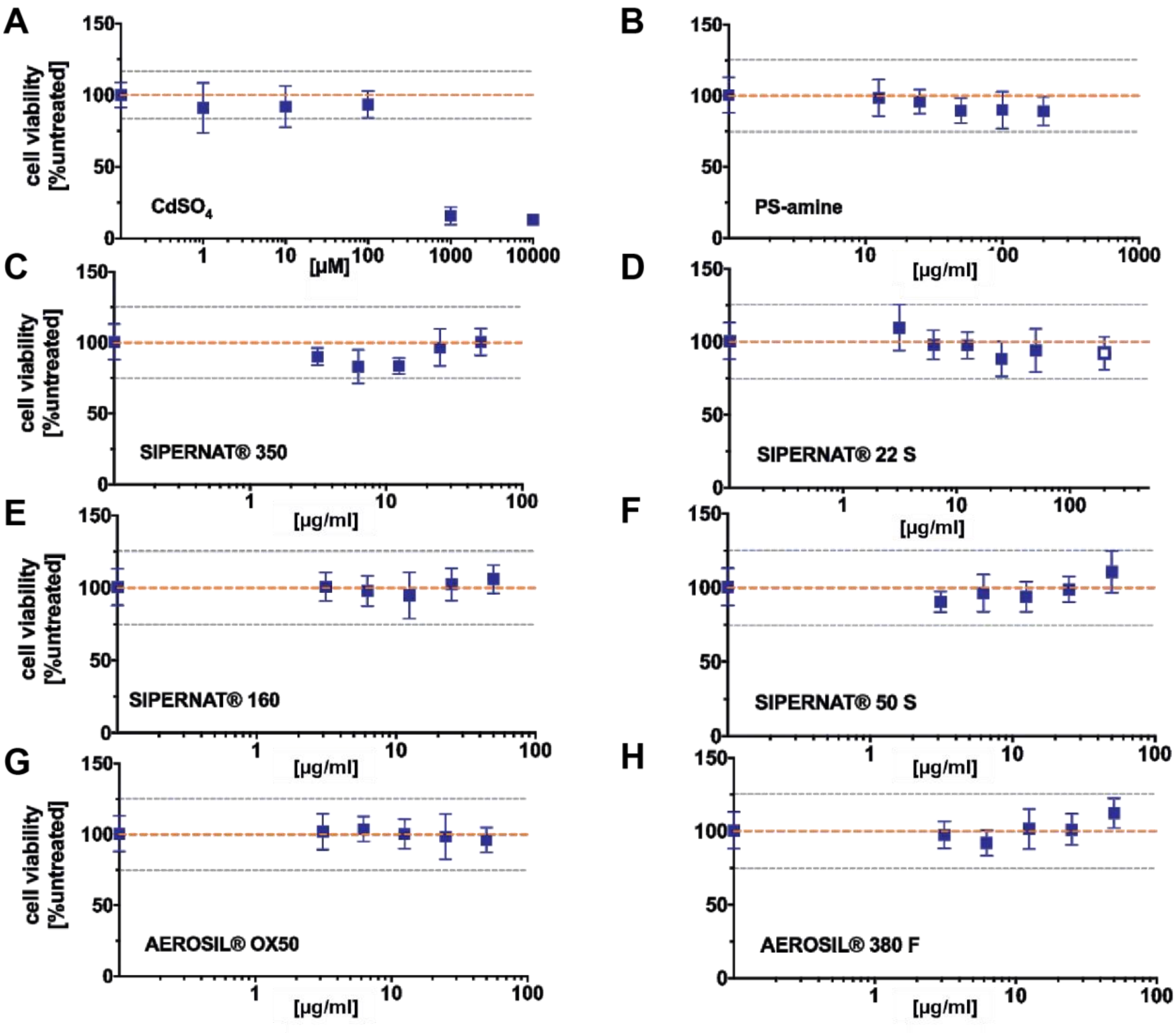

Figure S2: Impact of SAS on cell viability of advanced intestinal co-cultures after 24 h of exposure.

Following the incubation of advanced intestinal co-cultures with various concentrations of different SAS for 24 h cell viability was assessed with the MTT assay. (A) $CdSO_4$ served as a chemical positive control and (B) PS-amine was used as a particle positive control. (C) SIPERNAT® 350. (D) SIPERNAT® 22 S. Here also a higher concentration of 200 µg/ml (hollow symbol) was applied. (E) SIPERNAT® 160. (F) AEROSIL® OX50. (G) AEROSIL® 380 F. Mean values and corresponding standard deviations from three independent experiments with four technical replicates each are shown. The orange dashed line and the grey dotted lines resemble the mean value of the solvent control sample and twice the corresponding standard deviations, respectively.

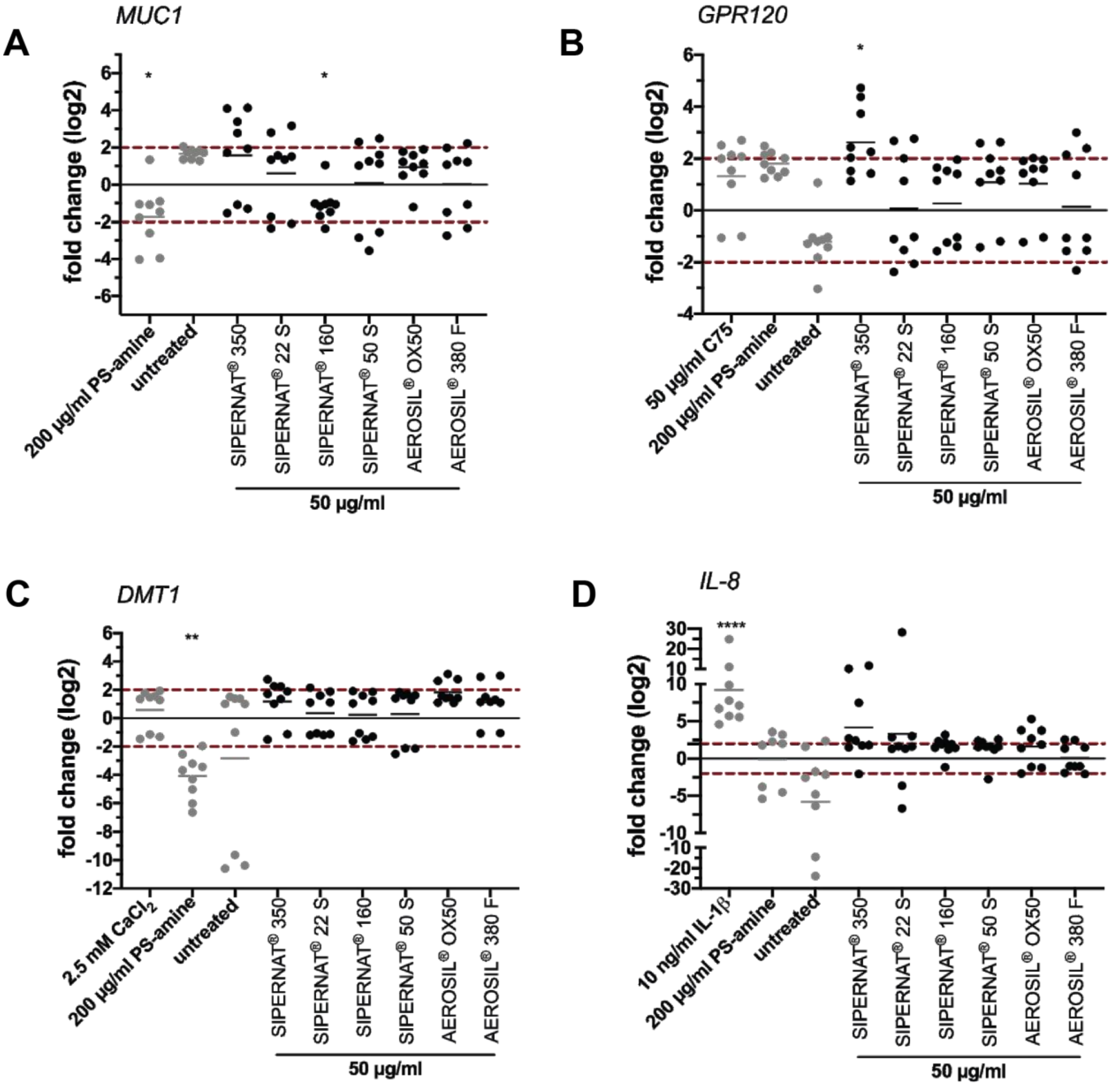

Figure S3: Impact of SAS on gene expression of *MUC1, GPR120, DMT1* and *IL-8* in advanced intestinal co-cultures after 24 h of exposure.

After 24 h incubation of advanced co-cultures with SAS, PS-amine or controls, the *MUC1* (A), *GRP120* (B), *DMT1* (C) *and IL-8* (D) gene expression was determined. The fold change of the gene expression has been compared to the solvent control. Shown are the normalised values to the solvent control and the housekeeping gene RLPLO of three independent experiments with three technical replicates. Mean values of all data points are shown as horizontal lines. The red dashed line represents gene expression of ±2. *P≤0.05, ****P≤0.0001 compared to the solvent control.

References

Antunes, F., Andrade, F., Araújo, F., Ferreira, D., & Sarmento, B. (2013). Establishment of a triple co-culture in vitro cell models to study intestinal absorption of peptide drugs. *European Journal of Pharmaceutics and Biopharmaceutics*, *83*(3), 427–435. https://doi.org/10.1016/j.ejpb.2012.10.003

Assimakopoulos, S. F. (2011). Enterocytes' tight junctions: From molecules to diseases. *World Journal of Gastrointestinal Pathophysiology*, *2*(6), 123. https://doi.org/10.4291/wjgp.v2.i6.123

Cabellos, J., Delpivo, C., Fernández-Rosas, E., Vázquez-Campos, S., & Janer, G. (2017). Contribution of M-cells and other experimental variables in the translocation of TiO2 nanoparticles across in vitro intestinal models. *NanoImpact*, *5*, 51–60. https://doi.org/10.1016/j.impact.2016.12.005

Campbell, J., Berry, J., & Liang, Y. (2019). *Chapter 71 – Anatomy and Physiology of the Small Intestine*. *Shackelford's Surgery of the Alimentary Tract, 2 Volume Set* (Eighth Edi). Elsevier Inc. https://doi.org/10.1016/B978-0-323-40232-3.00071-6

des Rieux, A., Fievez, V., Théate, I., Mast, J., Préat, V., & Schneider, Y.-J. (2007). An improved in vitro model of human intestinal follicle-associated epithelium to study nanoparticle transport by M cells. *European Journal of Pharmaceutical Sciences*, *30*(5), 380–391. https://doi.org/10.1016/j.ejps.2006.12.006

Hilgendorf, C., Spahn-Langguth, H., Regårdh, C. G., Lipka, E., Amidon, G. L., & Langguth, P. (2000). Caco-2 versus Caco-2/HT29-MTX Co-cultured Cell Lines: Permeabilities Via Diffusion, Inside- and Outside-Directed Carrier-Mediated Transport. *Journal of Pharmaceutical Sciences*, *89*(1), 63–75. https://doi.org/10.1002/(SICI)1520-6017(200001)89:1<63::AID-JPS7>3.0.CO;2-6

Lea, T. (2015). Caco-2 Cell Line. In *The Impact of Food Bioactives on Health* (pp. 103–111). Cham: Springer International Publishing. https://doi.org/10.1007/978-3-319-16104-4_10

Mabbott, N. A., Donaldson, D. S., Ohno, H., Williams, I. R., & Mahajan, A. (2013, July 22). Microfold (M) cells: Important immunosurveillance posts in the intestinal epithelium. *Mucosal Immunology*. https://doi.org/10.1038/mi.2013.30

Schimpel, C., Teubl, B., Absenger, M., Meindl, C., Fröhlich, E., Leitinger, G., … Roblegg, E. (2014). Development of an advanced intestinal in vitro triple culture permeability model to study transport of nanoparticles. *Molecular Pharmaceutics*, *11*(3), 808–818. https://doi.org/10.1021/mp400507g

Schneider, A., & Feussner, H. (2017). Anatomy, Physiology, and Selected Pathologies of the Gastrointestinal Tract. *Biomedical Engineering in Gastrointestinal Surgery*, 11–39. https://doi.org/10.1016/b978-0-12-803230-5.00002-6

Shearman, D. J. C., & Muir, A. R. (1960). OBSERVATIONS ON THE SECRETORY CYCLE OF GOBLET CELLS. *Quarterly Journal of Experimental Physiology and Cognate Medical Sciences*, *45*(4), 337–342. https://doi.org/10.1113/expphysiol.1960.sp001488

Shroyer, N. F., & Kocoshis, S. A. (2011). *Anatomy and Physiology of the Small and Large Intestines*. *Pediatric Gastrointestinal and Liver Disease* (Fourth Edi). Elsevier. https://doi.org/10.1016/B978-1-4377-0774-8.10031-4
